# Postural orthostatic tachycardia syndrome after radiofrequency catheter ablation in the atrioventricular junction—An uncommon and often unrecognized complication

**DOI:** 10.1016/j.hrcr.2024.06.003

**Published:** 2024-06-13

**Authors:** Dimitrios Lypourlis, Rakesh Agarwal

**Affiliations:** ∗ Cardiology Department, Lyell McEwin Hospital, Elizabeth Vale, Australia; † University of Adelaide, Adelaide, Australia

**Keywords:** Radiofrequency catheter ablation, Atrioventricular node, Postural orthostatic tachycardia syndrome, Intrinsic cardiac nervous system, Ivabradine

**Key Teaching Points**

• Radiofrequency catheter ablation in the vicinity of the atrioventricular (AV) junction may affect the intrinsic cardiac nervous system and be linked to postural orthostatic tachycardia syndrome (POTS).
• The diagnosis of POTS after radiofrequency catheter ablation may be delayed, as this condition is often not recognized despite debilitating symptoms.
• Awareness of the potential association of radiofrequency catheter ablation in the AV junction area and POTS may lead to its prompter diagnosis and management.
• Ivabradine is often used and is generally tolerated better than other alternatives. However, its efficacy may be limited, and it also may be associated with the occurrence of atrial fibrillation.

## Introduction

Despite atrioventricular nodal reentrant tachycardia (AVNRT) being one of the most common arrhythmias in clinical practice, its exact anatomic circuit remains elusive. Radiofrequency catheter ablation of the slow pathway has been used extensively for the treatment of AVNRT with a high success rate and overall low incidence of complications. The incidence of complete heart block as a result of radiofrequency ablation is under 1% among experienced operators. There has been a small number of reports of inappropriate sinus tachycardia but also postural orthostatic tachycardia syndrome (POTS) following radiofrequency ablation of AVNRT. We present a case of severe POTS after ablation in the proximity of the atrioventricular (AV) junction for atrial tachycardia.

## Case report

A 43-year-old female patient presented to the hospital with a 2-week history of rapid palpitations associated with mild light-headedness while standing up. These symptoms were so intense that she was forced to spend most of the day in bed. They started soon after a radiofrequency catheter ablation at a different hospital for what was thought to be an ectopic atrial tachycardia with a focus in the vicinity of the AV node. Before the ablation procedure, she had never experienced any similar symptoms. During the ablation procedure, she developed a complete heart block, and the procedure was abandoned. The AV block resolved within the following 6 hours. However, first-degree AV block with a PR interval of 220–240 ms remained thereafter.

**Figure 1:**
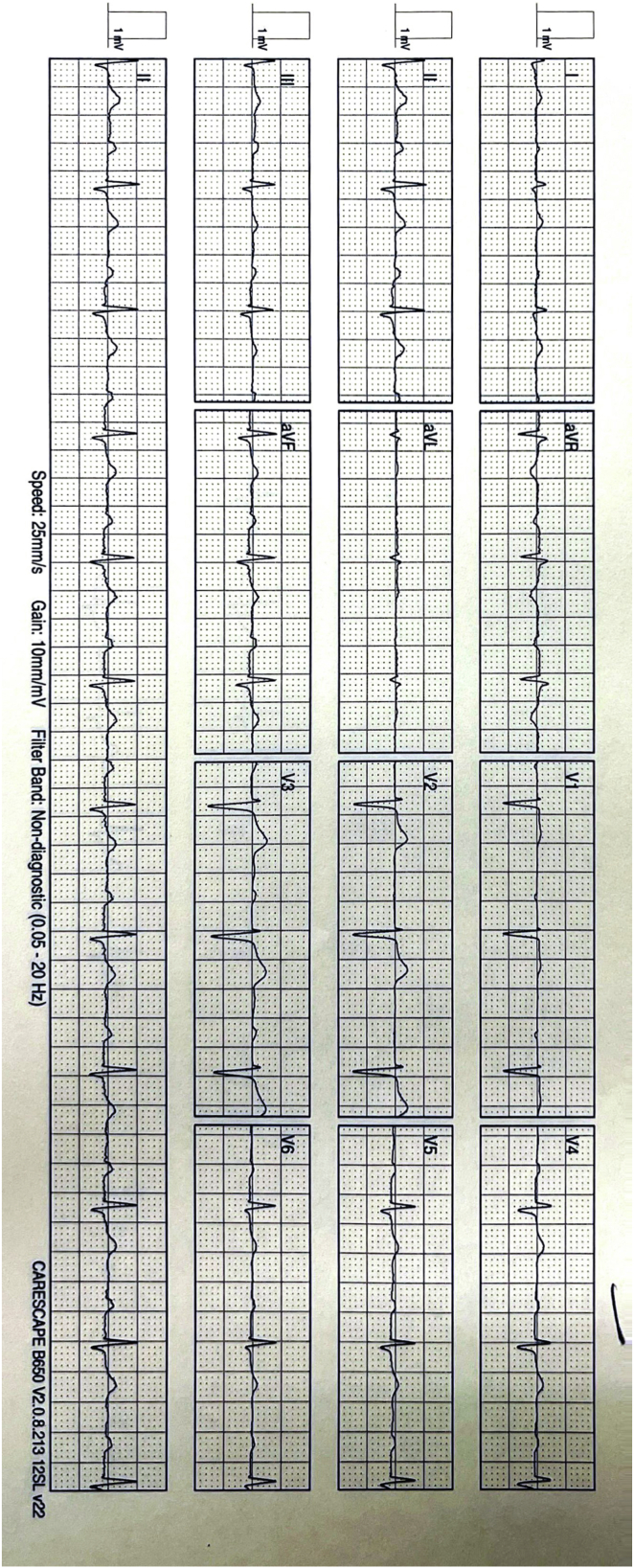
Resting electrocardiogram in supine position with prolonged PR interval.

**Figure 2:**
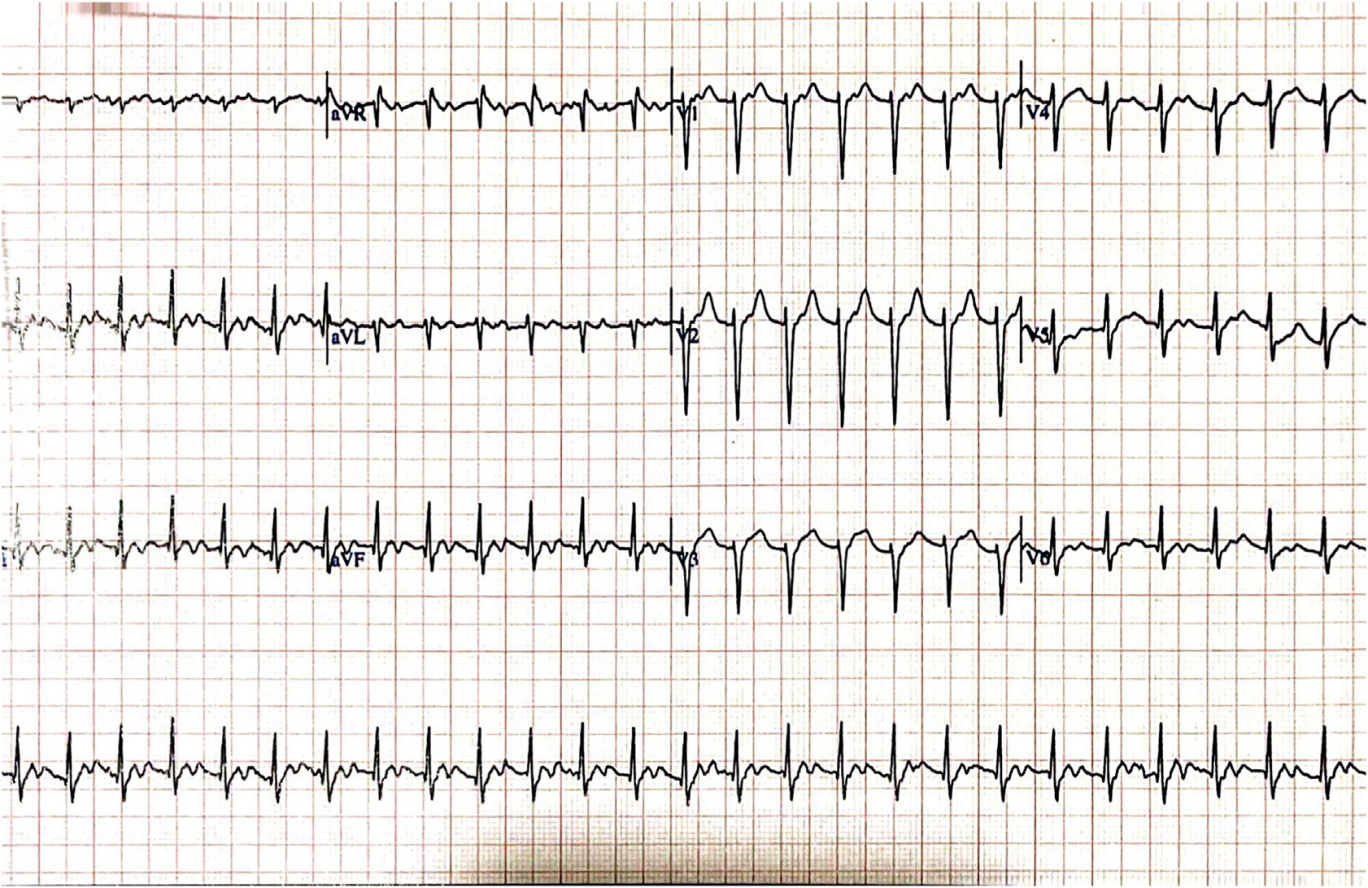
Immediate-onset tachycardia on upright posture.

**Figure 3:**
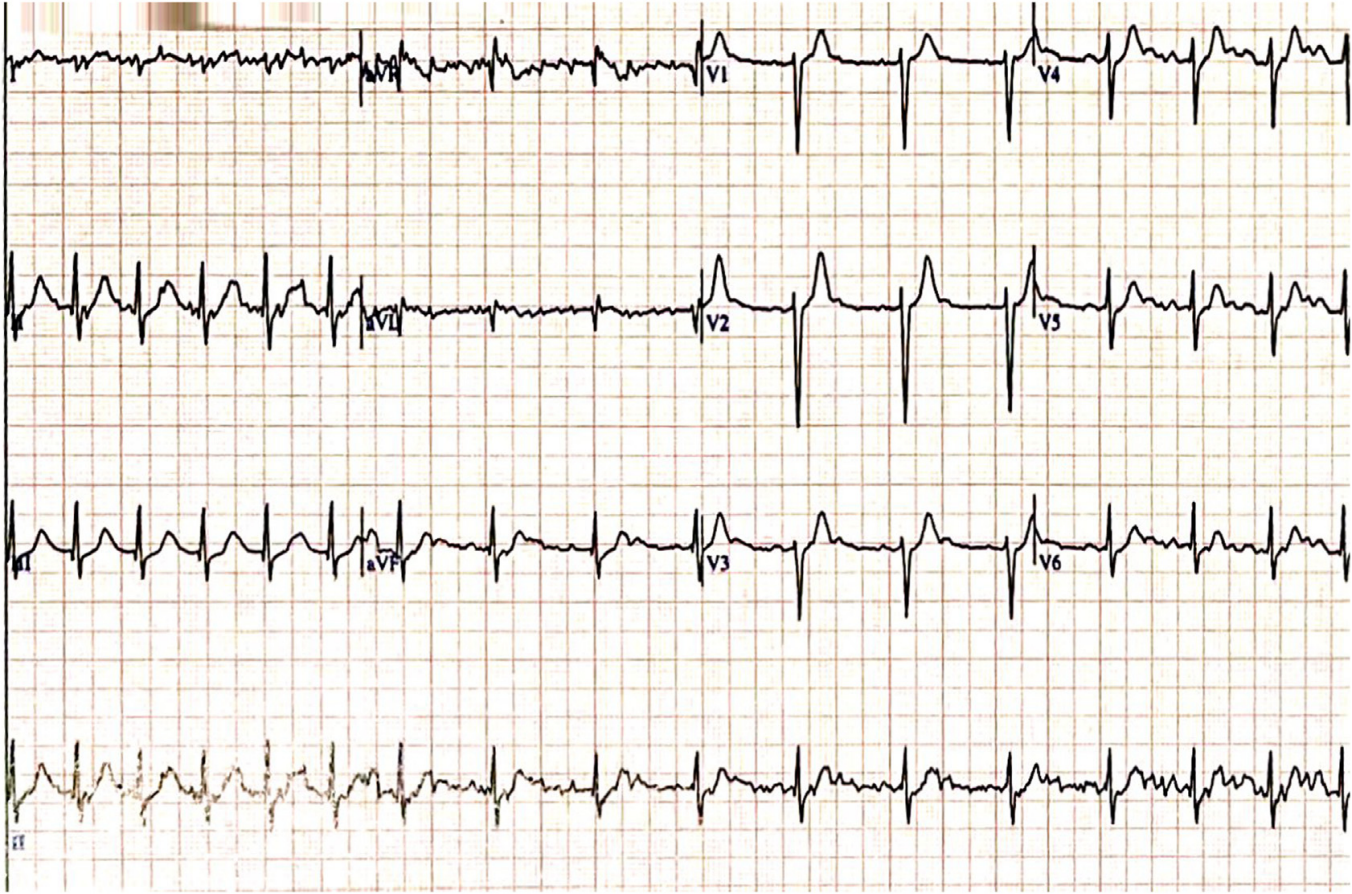
Electrocardiogram during recovery phase of exercise test with transient 2:1 atrioventricular block (best seen in leads V_1_–V_3_).

After her discharge from the hospital, she started to experience palpitations again; however, this time they felt entirely different and occurred invariably only after standing up. These were associated with nausea and light-headedness. She presented because of these symptoms to a different hospital, and she was discharged with a diagnosis of an ectopic atrial rhythm with concomitant high-grade AV block. She was then referred again to the electrophysiologist who had performed the ablation and was readmitted to the initial hospital for observation and cardiac monitoring. No arrhythmias or higher than first-degree AV block were recorded there while on telemetry. The treating physician attributed her complaints to anxiety and she was discharged after 3 days. Her symptoms, however, persisted and became debilitating over the following weeks, and she sought a second opinion. At the time of the consultation, a resting electrocardiogram in the supine position showed sinus rhythm with a heart rate of 67 beats per minute (bpm) and a PR interval of 240 ms (Figure 1). Within 1 minute of standing up, she started to experience her usual symptoms of palpitations associated with light-headedness and her heart rate increased to 160 bpm (Figure 2). Her blood pressure was 135/80 mm Hg and 130/80 mm Hg in the supine and upright positions, respectively. She managed to have a limited treadmill test to ascertain whether any high-grade AV block was present during exercise. She exceeded the target heart rate within the second minute of the first stage of the Bruce protocol without the occurrence of any high-grade AV block; however, the first-degree AV block persisted, and during the first minute of recovery, she had transiently 2:1 AV block of a few seconds’ duration (Figure 3). On assumption of the supine position, her heart rate gradually decreased to 80 bpm, and her symptoms subsided. After 10 minutes in the supine position and soon after standing up, her symptoms reoccurred with an increase in her rate from 80 bpm to 140 bpm and with no change in the blood pressure between the supine and upright position (140/80 mm Hg).

A diagnosis of POTS was made. The patient was advised to increase her fluid and salt consumption, and a trial of ivabradine at a starting dose of 5 mg twice daily was recommended, but she only agreed to have 2.5 mg twice owing to concerns that this might provoke a high-grade AV block. After having only 2 doses, she reported marked exacerbation of her palpitations, mainly at night time. She was therefore readmitted to the hospital for monitoring. Her nocturnal palpitations coincided with brief runs of atrial fibrillation. She refused to take any other medications because of fear that they may exacerbate her symptoms. She was discharged from the hospital with the advice to continue with increased fluid and dietary salt intake as well as aerobic exercise as tolerated. An implantable loop recorder was inserted, and home monitoring was set up. No further episodes of atrial fibrillation were recorded since the ivabradine was stopped. Furthermore, all the features of POTS gradually improved and essentially disappeared after 9 months.

## Discussion

POTS is a form of orthostatic intolerance associated with a significant increase in the heart rate (>30 bpm) within 10 minutes of assuming of the upright position and in the absence of orthostatic hypotension (blood pressure fall >20/10 mm Hg) and/or other overt causes of orthostatic tachycardia.

Despite similar clinical characteristics, POTS is likely a heterogeneous group of disorders. These include an autoimmune mechanism associated with antibodies to the postsynaptic acetylcholine receptors of the autonomic nerves, high levels of circulating norepinephrine as a result of a genetically determined malfunction of norepinephrine reuptake proteins, and importantly, a form of autonomic cardiac neuropathy. In a significant proportion of patients with POTS, however, no clear pathophysiology can be found. The onset of symptoms of POTS has been reported previously following electrocution^1^ and lightning injury.^2^

A report of 6 patients who developed debilitating POTS after immediately uncomplicated radiofrequency catheter ablation of the slow AV nodal pathway has been previously published.^3^ The exact frequency of POTS after radiofrequency ablation in the AV junction is unknown mainly owing to its generally low recognition. It is surprising that in the previous case report, none of the patients who developed POTS postablation were referred by the electrophysiologist who performed the ablation, as the patient’s symptoms were thought to be unrelated to the procedure and were dismissed as psychiatric, as was also initially the case with our patient.

The mechanism of POTS after radiofrequency ablation in the AV junction area is uncertain. Disruption of the vagal ganglia is a possible pathophysiological mechanism. It has been previously demonstrated that nerve fibers from both sides of both autonomic divisions project onto the AV node, although the differential effects of neural activity from the left and right sides are less clear. The density of cholinergic terminals appears to be considerably higher in the AV junction compared to other regions.^4^ Although the sympathetic innervation of the heart is extensive, the concentration of norepinephrine in the AV junction does not differ significantly compared to neighboring areas.^5^

Both the sinoatrial node (SAN) and the AV node have a rich supply of cholinesterase, with a ratio of cholinesterase distribution to nodal versus non-nodal tissue in the order of around 100:1. Normally, parasympathetic ganglia are not located within the AV node itself; however, they are at its posterior margin between the AV node and the anterior aspect of the coronary sinus ostium. It is this particular area that is targeted for radiofrequency ablation of the slow pathway, and disruption of the parasympathetic ganglia may affect the autonomic regulation of the SAN, leading to POTS.

The function of the intrinsic cardiac nervous system (ICNS) consisting of the ganglionated plexi (GP) and the atrial neural network is complex. The intrinsic cardiac nerve plexus (*GP and neural network*) acts as more than a simple relay station for extrinsic autonomic projections to the heart. It contains a heterogeneous population of cell types, including parasympathetic and sympathetic efferent neurons as well as afferent neurons, which participate in reflex loops between and within the various levels of the extrinsic, intrathoracic, and intrinsic systems. However, each level, when disconnected from the others, retains independent functionality.^6^ A GP in the intercaval region at the dorsal aspect of the right atrium was also shown to mediate vagal influences over the SAN.^7^ Anatomic studies in different mammalian species have consistently shown the presence of the right atrial GP with fibers originating in the epicardial fat pad and connecting to the SAN.^8^, ^9^, ^10^ These GPs have been shown to function not simply as relay stations made of postganglionic parasympathetic neurons but rather as interconnected integration centers.^6^

More recently, combining intersectional strategies, including tissue clearing, immunohistochemical, and ultrastructural techniques, a comprehensive neuroanatomic atlas of the right atrial GP and sinoatrial complex was delineated.^9^

Autonomic dysregulation may underlie many cases of POTS. Vagal nerve stimulation has been shown to cause a shift in the pacemaker site and activation pattern within the SAN region.^11^ Inadvertent abolition of parasympathetic input as a result of radiofrequency ablation would also be expected to affect the pacemaker site within the SAN, which may translate not only to a different sinus rate but also to different P-wave morphology, as was the case with our patient (Figures 2 and 3).

There has been some evidence that ablation of parasympathetic fibers may acutely increase the atrial refractory periods, followed by a decrease later, which may lead to an increase temporarily in the vulnerability to atrial fibrillation.^12^ This observation may also explain the often observed increase in the sinus rate transiently post plexus ablation with resolution after several months.

POTS is notoriously challenging to manage. Education to increase fluid and salt consumption, aerobic and resistance training, beta-blockers, midodrine, pyridostigmine, clonidine, methyldopa, selective serotonin reuptake inhibitors, or a combination has been used. Medication use, unfortunately, is often limited by unacceptable side effects. Ivabradine, an I_f_ channel blocker, is overall tolerated better. Ivabradine selectively blocks the I_f_ channels, which are responsible for spontaneous depolarization in the SAN, and regulates the sinus rate.

However, an increased incidence of atrial fibrillation has been reported in patients receiving ivabradine in the setting of acute coronary syndromes or heart failure.^11^ I_f_ current was also found in the pulmonary vein myocardial sleeves. Several studies showed that ivabradine may have a role in the maintenance of sinus rhythm^13^ owing to inhibition of the I_f_ current responsible for the ectopic activity arising in the pulmonary veins. The electrophysiological basis of ivabradine may also be linked to changes in the distribution of the channels that maintain the I_f_ current, the hyperpolarization-activated cyclic nucleotide-gated (HCN) cation channels. The presence of HCN4 channels in cardiac neurons may suggest that the I_f_ block ivabradine may exert its action via I_h_ in the ICNS in addition to affecting the I_f_ in the SAN.^14^ Alternatively, the transient atrial fibrillation in our patient early postablation may have been related to a transient change in the atrial refractory periods as a result of modulation of the parasympathetic input as a result of the radiofrequency current.^12^

## Conclusion

POTS may occur after ablation in the AV junction. The mechanism is unknown and possibly related to the modulation of the ICNS from the radiofrequency energy application. The exact incidence of this complication is unknown, mainly owing to its lack of recognition and under-reporting. Awareness of this may help in more prompt diagnosis and management of this often debilitating complication.

## Disclosures

Dr Agarwal is an editorial board member of *Heart Rhythm Case Reports*. Dr Lypourlis has no conflicts to disclose.
